# Automated 3D Segmentation of the Aorta and Pulmonary Artery on Non-Contrast-Enhanced Chest Computed Tomography Images in Lung Cancer Patients

**DOI:** 10.3390/diagnostics12040967

**Published:** 2022-04-12

**Authors:** Hao-Jen Wang, Li-Wei Chen, Hsin-Ying Lee, Yu-Jung Chung, Yan-Ting Lin, Yi-Chieh Lee, Yi-Chang Chen, Chung-Ming Chen, Mong-Wei Lin

**Affiliations:** 1 Department of Biomedical Engineering, College of Medicine and College of Engineering, National Taiwan University, Taipei 106, Taiwan; d04548013@ntu.edu.tw (H.-J.W.); f04548034@ntu.edu.tw (L.-W.C.); r08548005@ntu.edu.tw (Y.-J.C.); r08548047@ntu.edu.tw (Y.-T.L.); scsnake@gmail.com (Y.-C.C.); 2 Department of Medicine, National Taiwan University, Taipei 100, Taiwan; b04401128@ntu.edu.tw (H.-Y.L.); b06401027@ntu.edu.tw (Y.-C.L.); 3 Department of Surgery, National Taiwan University Hospital and National Taiwan University College of Medicine, Taipei 100, Taiwan

**Keywords:** aorta, computed tomography, deep learning, lung cancer, pulmonary artery, pulmonary hypertension

## Abstract

Pulmonary hypertension should be preoperatively evaluated for optimal surgical planning to reduce surgical risk in lung cancer patients. Preoperative measurement of vascular diameter in computed tomography (CT) images is a noninvasive prediction method for pulmonary hypertension. However, the current estimation method, 2D manual arterial diameter measurement, may yield inaccurate results owing to low tissue contrast in non-contrast-enhanced CT (NECT). Furthermore, it provides an incomplete evaluation by measuring only the diameter of the arteries rather than the volume. To provide a more complete and accurate estimation, this study proposed a novel two-stage deep learning (DL) model for 3D aortic and pulmonary artery segmentation in NECT. In the first stage, a DL model was constructed to enhance the contrast of NECT; in the second stage, two DL models then applied the enhanced images for aorta and pulmonary artery segmentation. Overall, 179 patients were divided into contrast enhancement model (n = 59), segmentation model (n = 120), and testing (n = 20) groups. The performance of the proposed model was evaluated using Dice similarity coefficient (DSC). The proposed model could achieve 0.97 ± 0.007 and 0.93 ± 0.002 DSC for aortic and pulmonary artery segmentation, respectively. The proposed model may provide 3D diameter information of the arteries before surgery, facilitating the estimation of pulmonary hypertension and supporting preoperative surgical method selection based on the predicted surgical risks.

## 1. Introduction

Low-dose computed tomography (CT) screening has recently increased the detection rate of early-stage lung cancer [1,2,3]. Thoracic surgical resection is the major treatment approach for patients with early-stage lung cancer [4,5,6,7]. Surgical planning may vary from patient to patient owing to different surgical risks across patients. Extensive resection (lobectomy) is the treatment of choice for patients with a low surgical risk and high tumor invasiveness. However, limited resection (wedge resection or segmentectomy) is indicated for patients with high surgical risks and low tumor invasiveness [8,9,10,11]. Preoperative pulmonary hypertension associated with postoperative heart failure has been indicated to be exacerbated by surgery, leading to an increase in mortality risk (four to five times higher than that in patients without pulmonary hypertension) [12,13]. Furthermore, Wei et al. showed that the failure rate of the right ventricle was significantly higher in patients with pulmonary hypertension before surgery (10.5%) than in patients without pulmonary hypertension (2.2%) [14]. Therefore, pulmonary hypertension is a surgical risk factor that may result in malignant behaviors; thus, it is important to preoperatively evaluate the presence of pulmonary hypertension, supporting surgical management. The gold standard approach for the diagnosis of pulmonary hypertension is the direct measurement of pulmonary artery pressure by cardiac catheterization [12,14]. However, this invasive measurement method may not be commonly used for the preoperative evaluation of patients with lung cancer. Cardiac ultrasound examination before lung cancer surgery would be an alternative approach to confirm the presence of pulmonary hypertension before surgery [12,14]. However, these results are unreliable and lack accuracy.

Several previous studies have indicated that the diameter of the pulmonary artery, or the ratio of the pulmonary artery to the aorta, is an effective tool for assessing pulmonary hypertension [4]. Chung et al. published imaging studies that measured these two parameters found that the diameter of the pulmonary artery increased significantly after lobectomy (23.9–25.6 mm, *p* < 0.0001) [15]. However, the method used in that study was a 2D measurement of contrast-enhanced chest CT images. As the majority of patients with lung cancer are diagnosed during low-dose CT (LDCT) screening, these patients may not have undergone contrast-enhanced CT before surgery. In addition, post-surgery tracking is usually by non-contrast-enhanced CT, and the technology of image measurement based on non-contrast-enhanced CT still needs to be developed.

The present study aimed to develop an automatic 3D segmentation method for the aorta and pulmonary artery on non-contrast-enhanced CT images to accurately calculate the 3D diameter information of the two arteries before surgery, facilitate the estimation of pulmonary hypertension, and support preoperative surgical management.

## 2. Materials and Methods

### 2.1. Data Information

Preoperative chest CT images of 179 patients with lung cancer were collected from the National Taiwan University Hospital between January 2011 and December 2019, and all patients had a set of CT images without a contrast agent and with a contrast agent. The inclusion criteria of the study were as follows: (1) pathologically confirmed lung cancer, and (2) available thin-cut chest CT image data. The Research Ethics Committee of the National Taiwan University Hospital approved this study (project approval number 201712087RIND) and waived the need for informed consent because of the retrospective study design.

**Figure 1:**
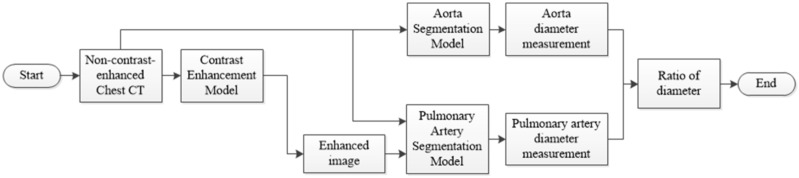
Flowchart of the pulmonary hypertension assessment method.

The overall flowchart of the pulmonary hypertension assessment method is shown in Figure 1. In this study, two types of models, namely, the contrast-enhanced and segmentation models, were developed to achieve segmentation of the aorta and pulmonary artery in two stages. CT images obtained with a contrast agent and without a contrast agent were used in the contrast-enhanced model. The training data were allocated to 49 patients, and the validation data were allocated to 10 patients, with a total of 59 patients. On the contrary, because the clinical application of this model presupposes that patients are not evaluated with a contrast agent, the data used for the segmentation model were taken from non-contrast-enhanced CT images. In the segmentation model, 120 non-contrast-enhanced CT images from the dataset were used, divided into 80 patients (training data), 20 patients (validation data), and 20 patients (testing data), and this configuration was used for segmentation model training.

### 2.2. CT Image Acquisition

Chest CT scans used in this study were acquired from the following manufacturers using a multidetector (16-, 32-, or 64-detector row) CT scanner: GE (LightSpeed VCT, LightSpeed 16, and HiSpeed CT/I, Chicago, IL, USA), Siemens (Definition AS+, Emotion 16, and Sensation 64, Erlangen, Germany), and Philips (iCT 256 and Ingenuity CT, Amsterdam, Netherlands) Healthcare systems. The CT image parameters were as follows: detector collimation, 0.6–1.25 mm; field of view, 20–38 cm; beam pitch, 0.800–1.396; beam width, 10–40 mm; gantry speed, 0.5 or 0.8 s per rotation; 100–130 kVp; 47–351 mA; reconstruction interval, 0.39–6 mm; matrix, 512 × 512 mm^2^.

### 2.3. Pre-Processing of CT Images

**Figure 2:**
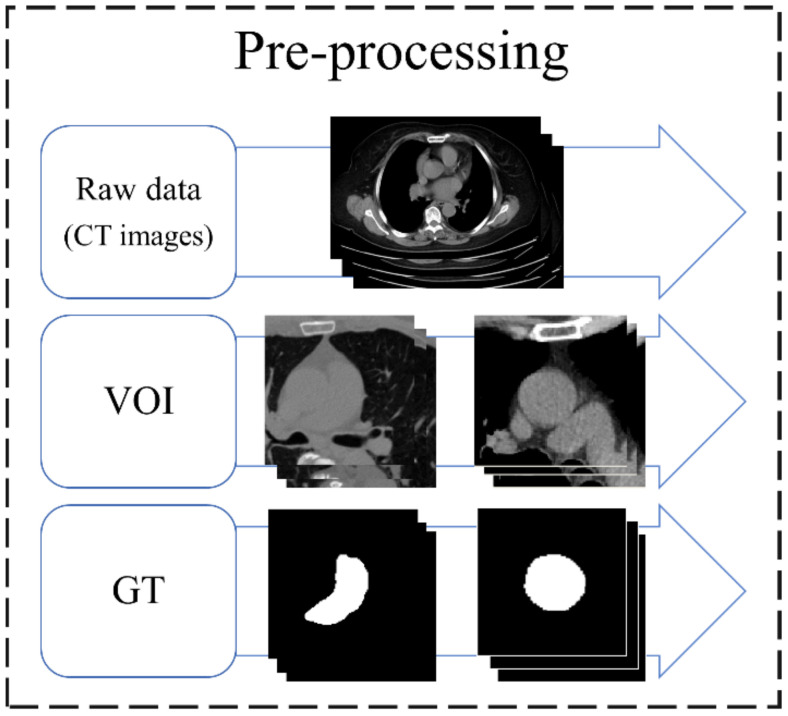
Preprocessing flow of data. VOI, volume of interest; GT, ground truth.

The original image was a set of chest CT Dicom images, and each data were resampled to 0.1 mm in data preprocessing, and the value was between −160 and 240 HU. To ensure that the deep learning (DL) model learns the location of blood vessels accurately, the aorta and pulmonary artery are considered as the center to cut out two sizes of volume of interest (VOI) (96 × 96 × 32 and 128 × 128 × 64). The aorta requires a larger VOI because of its anatomical shape that covers more slices. As there is currently no commercial software that can accurately annotate the aorta and pulmonary artery for CT images without contrast agents, this study invited two professional thoracologists to assist in the annotation of the ground truth (GT) of the two targets in this study. The preprocessing flow of the data is shown in Figure 2.

### 2.4. Architecture

**Figure 3:**
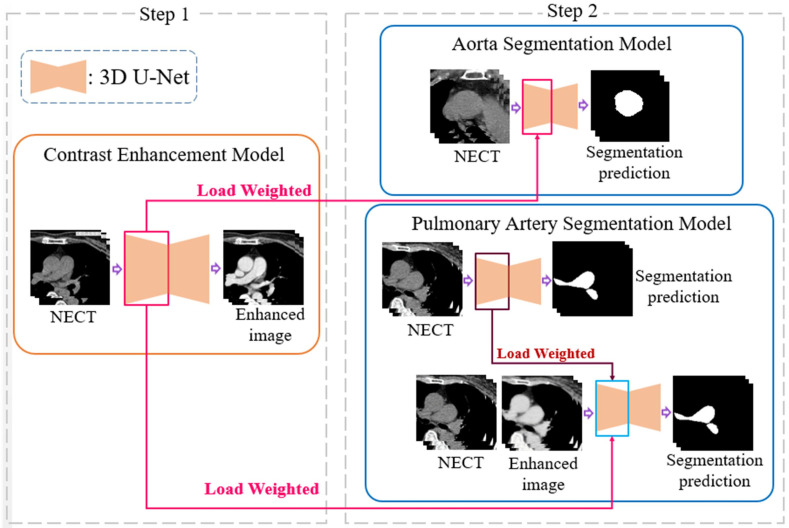
A two-stage deep learning architecture for 3D segmentation. NECT, non-contrast-enhanced Chest CT.

To evaluate the complications, measurements of the diameter of the aorta and pulmonary artery and calculation of the ratio are required. This study proposes a two-stage DL architecture for the 3D segmentation of blood vessels. Because this study used non-contrast-enhanced CT images, a contrast enhancement model was used in the first stage to enhance the non-contrast images of the aorta and pulmonary artery; thus, the contrast between the blood vessels and the surrounding tissues in the non-contrast-enhanced CT images was improved. This model increases the sharpness of the blood vessel edge to facilitate the effective learning of the backward segmentation model. In the second stage, two 3D vessel segmentation models were developed for the aorta and pulmonary artery, as shown in the flowchart (Figure 3). After successfully segmenting the two vessels, the vessel sections were extracted to obtain the average diameter of the 3D vessel. The overall architecture is shown in Figure 3.

### 2.5. 3D U-Net

**Figure 4:**
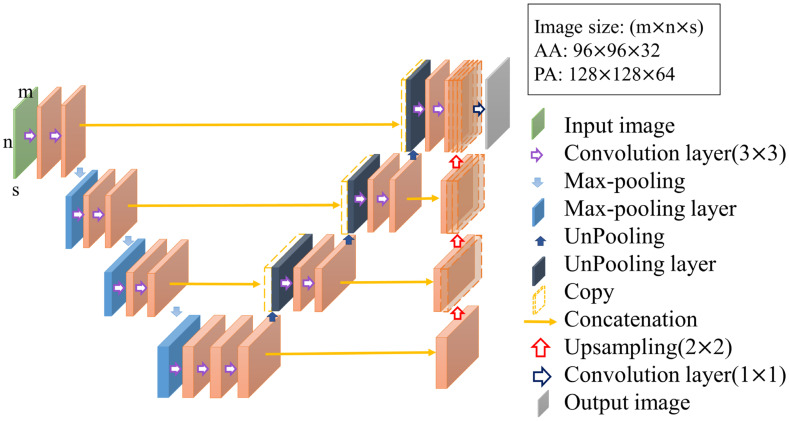
Structure of 3D U-Net.

The contrast enhancement model and segmentation model proposed in this study are improved models based on U-Net [16,17]. U-Net is a convolution-based model that can be modeled by point-by-point convolution and superimposed on each convolution layer. It is a type of fully convolutional network (FCN) model [18] and is composed of a downsampling (contraction) path to aggregate high-level information using context modules and an upsampling (expansion) pathway to combine feature and spatial information for localization. In the downsampling path, each layer consists of two 3 × 3 convolutional layers, and then downsampling with a stride of 2 is used to extract information and capture the contour features of the input image with missing spatial information. This gradually restores the image size through upsampling with a step size of 2, extracting information on important features from the original image information and integrating contextual information. Therefore, this model can perform feature extraction and multi-information transmission through two paths to achieve semantic segmentation. The network used in this study, called 3D U-Net, was changed from its original 2D architecture to a 3D architecture by using 3D volumes as input and processing them with corresponding 3D operations, such as 3D convolutions and 3D max pooling, as shown in Figure 4 [19].

### 2.6. Contrast Enhancement Model

**Figure 5:**
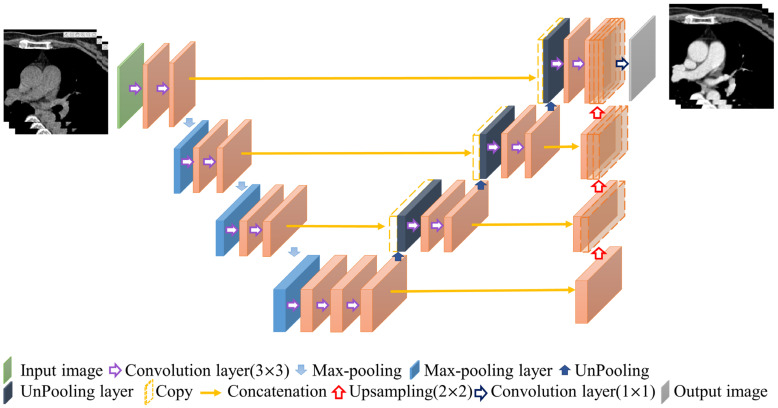
Contrast enhancement model.

In non-contrast-enhanced images, because the image contrast is not significant and the blood vessel boundaries are relatively blurred, it is not easy to segment the blood vessels directly. Therefore, this study proposes a contrast-enhancement model that uses the corresponding non-contrast- and contrast-enhanced images as the input and GT of the model, respectively, as shown in Figure 5. This model learns how to generate contrast-enhanced images from non-contrast-enhanced images so that the second-stage model in the architecture can more easily achieve segmentation. In this model, a combination of mean absolute error (MAE) and structural dissimilarity (DSSIM) loss functions is used as the loss function [20,21]. The MAE is the sum of the absolute values of the difference between the target value and the predicted value. It measures only the average error of the predicted value, regardless of the direction, and ranges from 0 to positive infinity; therefore, it can be used to judge the overall contrast enhancement performance of this model. DSSIM was derived from a formula based on the structural similarity index (SSIM) [21,22]. SSIM combines luminance, contrast, and structure to reflect the structural information heavily relied on by anthropology. In the chest CT images used in this study, there is a strong correlation between adjacent pixels in the same anatomical structure; therefore, it is suitable for use as the loss function of this model [22]. Therefore, this study adopts the advantages of the two loss functions and sets the trade-off parameter α to the optimal value of 0.7 after many experiments in this study, as shown in Formula (1), where I_gt and I_op are the GT and model output, respectively. In this study, this loss function is used in a U-Net model with the ability to integrate contextual information for contrast enhancement model training.

(1) $$Loss\left(I\_op,\;\;I\_gt\right)=\alpha MAE\left(I\_op,\;\;I\_gt\right)+\left(1-\alpha \right)DSSIM\left(I\_op,\;\;I\_gt\right)$$

### 2.7. Segmentation Model

In the second stage of the architecture, this study developed relative 3D vessel segmentation models for two vessels, namely the aorta and pulmonary artery. The ratio of the diameter of the aorta to that of the pulmonary artery is an important indicator of the presence or absence of pulmonary hypertension. To obtain the diameters of the two blood vessels, this study developed a 3D segmentation model to overcome the disadvantage of non-contrast-enhanced CT images to segment the anatomical structures of the two blood vessels and then extract the blood vessel sections to calculate the average blood vessel diameter.

#### 2.7.1. Aorta Segmentation Model

**Figure 6:**
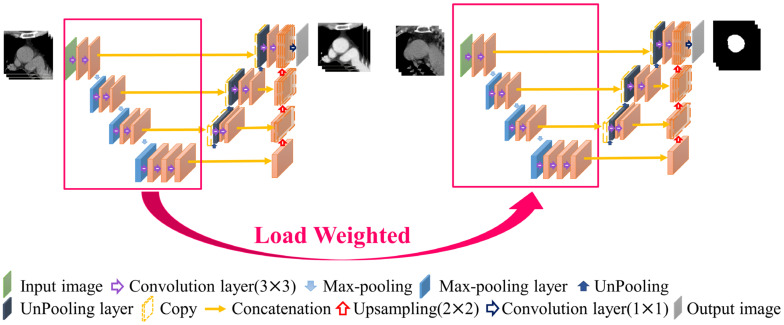
Aorta segmentation model.

The training process of the contrast enhancement model also learns the difference between the voxels of the blood vessels and those of other thoracic anatomical structures, which is similar to the purpose of finding the position of the blood vessel boundary in the segmentation model. Therefore, the training weights obtained in the first stage of the architecture are suitable for transfer learning to improve the learning efficiency of the segmentation model [23]. The location and method of the weights used in the transfer learning are shown in Figure 6. The aorta is relatively simple in the thoracic structure; therefore, this study directly uses unenhanced CT images for training and combines the loss function of the Dice loss function commonly used in segmentation models to achieve the training and development of the aorta segmentation model, in which both Pi and Gi are a single voxel of GT and model output, respectively, and N is the total number of voxels of the data, as shown in Formula (2).

(2) $$\mathrm{DSC}=\frac{2\sum_{i}^{N}P_{i}g_{i}}{\sum_{i}^{N}p_{i}^{2}+\sum_{i}^{N}g_{i}^{2}}$$

#### 2.7.2. Pulmonary Artery Segmentation Model

**Figure 7:**
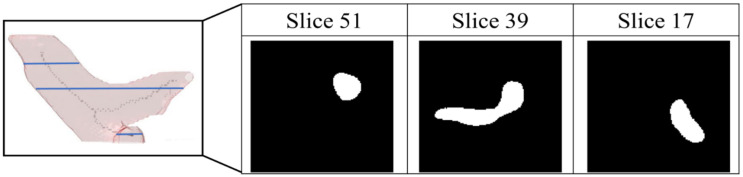
Pathological shape images of pulmonary aorta in different slice of the computed tomography images.

**Figure 8:**
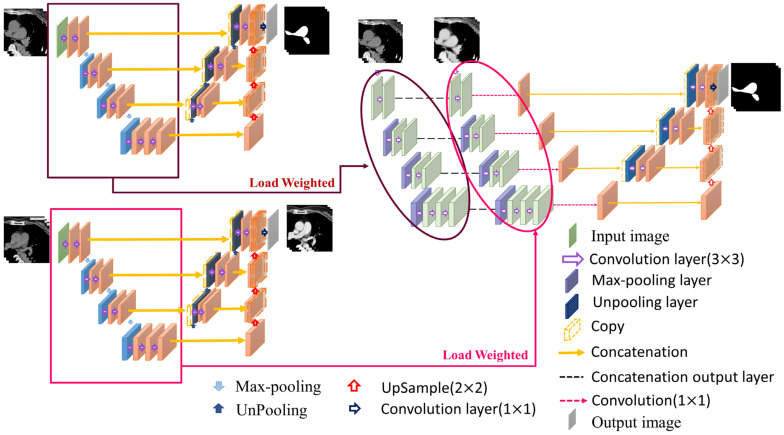
Pulmonary artery segmentation model.

The pathological structure of the pulmonary artery is relatively variable in the direction and shape of the blood vessels. From the cross-section of the CT image, it can be observed that the shape of each slice is very different (Figure 7). To enable the model to learn the target more accurately, this study designed this model as a two-channel model. In addition to the original non-contrast-enhanced CT image as the input, the contrast-enhanced image learned by the contrast enhancement model is used as the second channel to input the pulmonary artery segmentation model. In addition, consistent with the aorta segmentation model, this model also uses transfer learning for the augmentation training of the model. The difference is that for the model to learn more accurate pulmonary artery voxel information, the model will pre-train the segmentation model and then concatenate the weights obtained in this pre-training with the training weights in the contrast enhancement model. Based on this, transfer learning was performed on the pulmonary artery segmentation model, as shown in Figure 8, to achieve a more complete pulmonary artery segmentation result.

In this segmentation model, the combination of the weights obtained by segmentation pre-training and the training weights in the contrast enhancement model is shown in Figure 8. In this study, each layer of downsampling was combined individually to ensure that, during the feature extraction stage, both channels maintained the training impact. The model hyperparameters used in this study are presented in Table 1.

### 2.8. Vessel Diameter Measurement

**Figure 9:**
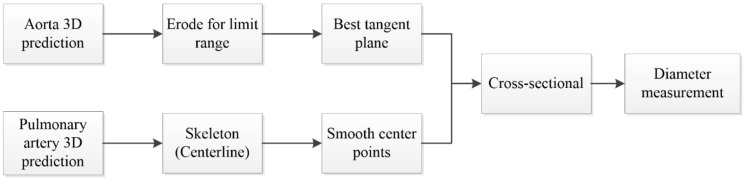
Flowchart of diameter measurement.

After obtaining the aorta and pulmonary artery from the two-stage segmentation architecture, this study developed a mean diameter measurement method for both vessels for the vessel diameter measurement segment required for assessing pulmonary hypertension. To measure the vessel diameter, this study determined the centerline from the segmented 3D vessel images. Find the corresponding blood vessel section by the point on the centerline and calculate its diameter. After summation and averaging, the average diameters of the two blood vessels were obtained. However, the vascular shape of the pulmonary artery is more tortuous than that of the aorta; therefore, two different methods were used in the anterior segment of the measurement process (Figure 9).

**Figure 10:**
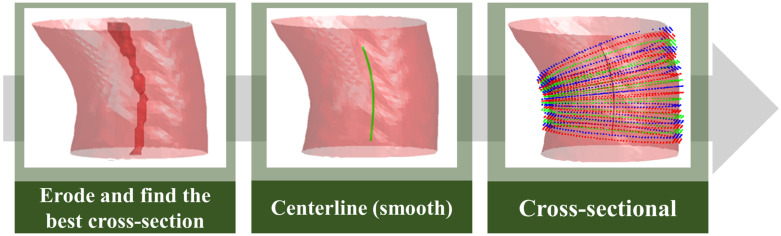
Aorta diameter measurement method.

According to the characteristics of the aorta blood vessel itself, the blood vessel was measured from 0.5 cm after exiting the heart to the position of 2.5 cm, as the range for calculating the average diameter of the entire aorta. The blood vessel diameter was measured (Figure 10). The original three-dimensional blood vessel is eroded to obtain a region as a limiting range to find the center line; second, the direction vector between points and the blood vessel surface are used. The normal vector is used as the inner product, and the minimum inner product value between each candidate point is compared to select the next point and so on until the entire blood vessel is searched; finally, the diameter of the blood vessel section perpendicular to the centerline of each segment is used to calculate the average diameter.

**Figure 11:**
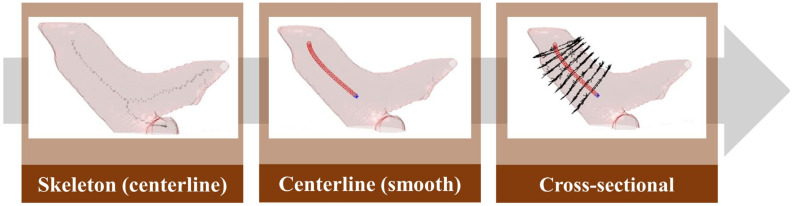
Pulmonary artery diameter measurement method.

To obtain the average diameter of the pulmonary artery, this study first used skeletonization [24] to determine the rough centerline (Figure 11) and started to measure along the main vessel of the pulmonary artery 0.5–1.5 cm from the position of the branch points. Furthermore, the points on the centerline were discretized by interpolation. This step smoothens the centerline. Finally, the diameter of the blood vessel section perpendicular to each segment of the centerline was calculated at every 0.04-cm interval to obtain the average diameter of the pulmonary artery.

## 3. Results

### 3.1. Patient Clinicopathological Features and Perioperative Results

This study cohort comprised 179 patients diagnosed with lung cancer who underwent lobectomies between 2011 and 2019. The mean age of all the 258 patients was 78.6 ± 3.3 years (range: 75−90). The majority of patients were females (64.2%) and non-smokers (78.8%). The mean postoperative intensive care unit stay and hospital stay were 0.3 and 5.3 days, respectively. There was no 30-day mortality in the study cohort. Patient clinicopathological features and perioperative results are presented in Supplementary Table S1.

### 3.2. Contrast Enhancement Model

**Figure 12:**
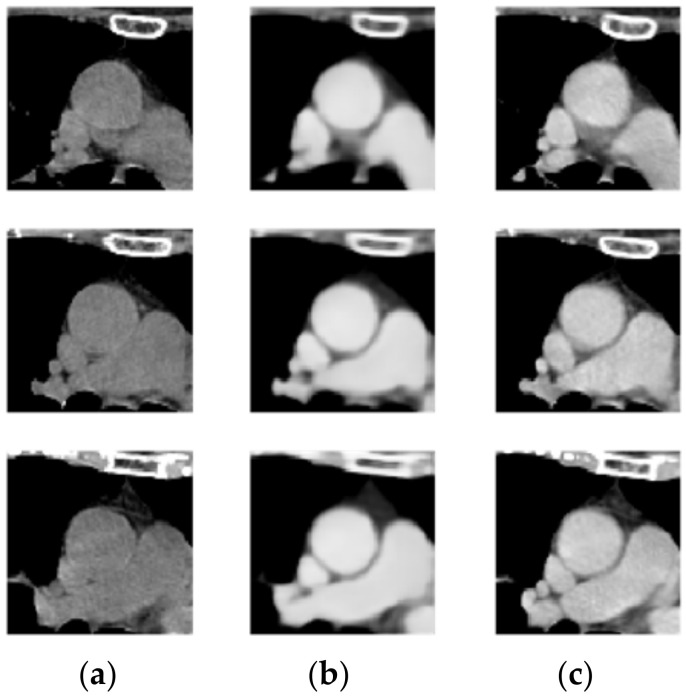
(**a**) Non-contrast-enhanced chest CT, (**b**) enhanced image, and (**c**) contrast CT image.

In the first stage of the architecture, there was only a slight difference between the contrast enhancement generated by the non-contrast agent CT image and the real contrast agent CT image. The method developed in this study can significantly enhance the vascular contrast of the non-contrast agent CT image, as shown in Figure 12.

### 3.3. Segmentation Model

**Figure 13:**
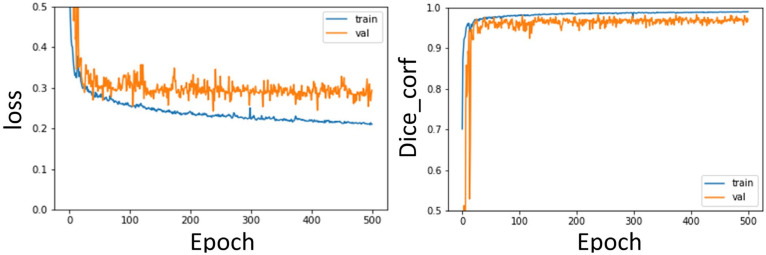
Aorta segmentation model training curve: Loss, Dice curve.

**Figure 14:**
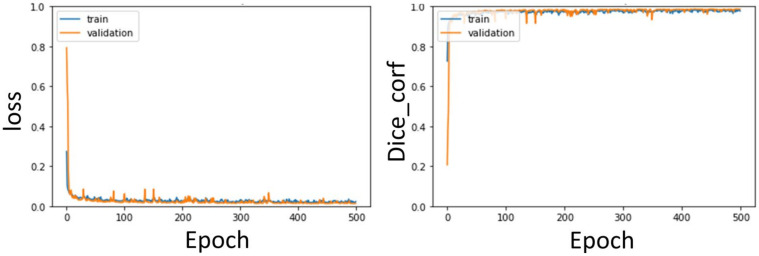
Pulmonary artery segmentation model training curve: Loss, Dice curve.

The training curves of the segmentation model of the aorta and pulmonary artery in this study are shown in Figure 13 and Figure 14. It can be seen that regardless of whether it is the segmentation of the aorta or the segmentation model of the dual-channel pulmonary artery, in the later stage of model training, reliable and stable results can be achieved for both the training dataset and the validation dataset.

In the second stage of the architecture, the weights of the first stage are utilized by transfer learning, which can improve the learning performance of the model. Therefore, the segmentation results of the aorta and pulmonary artery by the method proposed in this study can achieve Dice coefficients of 0.97 ± 0.007 and 0.93 ± 0.002, respectively, after fivefold cross-validation.

In addition, this study compared the results of the proposed model with the unimproved 3D U-Net mentioned in Section 2.5, as shown in Table 2.

From the results, the two-stage DL segmentation model proposed in this study can efficiently complete the three-dimensional segmentation of the two major blood vessels, and for the difficult pulmonary artery, additional input imaging enhanced images can effectively improve segmentation performance. Among them, the pulmonary artery segmentation model adds contrast-enhanced images as the second channel, and it can be seen from the segmentation results that its performance is better than that of the single-channel input of only non-contrast-enhanced CT images. As shown in Table 2, the method proposed in this study is far superior to the 3D U-Net in either aortic segmentation or pulmonary artery segmentation.

## 4. Discussion

This study used fivefold cross-validation and DSC as the evaluation metrics, and the results are shown in Table 2. For aorta segmentation, the performance of this segmentation model was 0.97 ± 0.007, and it was only required to input non-contrast-enhanced CT images, which was in line with clinical use. Pulmonary artery segmentation is more difficult than aorta segmentation because of its complex vessel orientation. As shown in Table 2, the result of this model is 0.91 ± 0.002 when inputting only non-contrast-enhanced CT images, which is relatively poor. Therefore, this study adds the contrast-enhanced images obtained in the first stage of the architecture to improve segmentation performance. The result of this two-channel pulmonary artery segmentation model is 0.93 ± 0.002, which is approximately 0.02 higher than the input of only non-contrast-enhanced CT images.

To verify the effectiveness of the two-stage method in this study, we compared the segmentation performance of several previous studies, as shown in Table 3 [25,26,27,28,29,30,31,32,33,34,35,36,37,38,39]. In terms of aorta segmentation, the method proposed in this study achieved the highest segmentation performance, whereas in terms of pulmonary artery segmentation, it was only slightly inferior to the method developed by Gamechi et al. The method used in this study still has a high-precision segmentation performance.

In the past, research on segmentation of the aorta and pulmonary artery on CT images has been conducted for many years. Therefore, this study also compared the performance of a previous study with that of the method used in this study. Previous studies on aorta segmentation mostly used images taken with a contrast agent for algorithm development. Compared with images taken with a non-contrast agent, those taken with a contrast agent has better vascular contrast and the vascular lumen presents a more obvious grayscale contrast with the surrounding tissue. Therefore, blood vessel segmentation is easier to perform. Jang et al. used CT images of contrast agents and proposed a method of automatic segmentation of the ascending aorta using geodesic distance transformation combined with Hough circles, which was applied to the diagnosis of cardiovascular diseases [25]. The proposed method outperforms this method in terms of segmentation performance based on non-contrast images; therefore, it is more competitive.

CT screening for early detection of thoracic cavity disease was performed without contrast agents. In addition, contrast injections should not be used in patients with allergy to these contrast media. Therefore, in recent years, most studies have been conducted on CT images without the use of contrast agents. In studies on aorta segmentation on non-contrast images, the following methods have been proposed based on prior knowledge of the vessel shape [26,28,29,30,32]. Isgum et al. proposed a multiple atlas-based segmentation method that registers multiple manually segmented atlas to the target image and uses decision fusion to obtain segmentation results [26]. Avila-Montes et al. proposed the extraction of the aorta centerline by Hough transform and dynamic programming and used the entropy-based cost function for boundary detection [28]. Dasgupta et al. used the circular Hough transform method to locate the vessel region to obtain aorta segmentation results [29]. Xie et al. proposed an algorithm that uses anatomy label maps and cylinder tracking to segment the aorta [30]. Gamechi et al. combined multi-atlas registration to obtain seed points, aorta centerline extraction, and optimal surface segmentation to extract the aorta in non-contrast-enhanced CT images [32]. However, the extraction of the aorta centerline or boundary based on such shape priors is prone to errors in the locations where some vessels are narrowed, dilated, or where plaques appear.

In a study based on the active contour [40] method to segment the aorta, Kurugol et al. used the Frenet framework and 3D level set method to develop a fully automated and unsupervised segmentation of the aorta. The Dice coefficient was 0.93 ± 0.01. The aorta segmentation results can be used to quantify the degree of aorta calcification [27]. Kurugol et al. exploited the cross-sectional circularity of the aorta in axial slices and the aortic arch in reformatted oblique slices to detect initial aorta boundaries and used the 3D level-set method to modify the final results. The efficacy yields a Dice coefficient of 0.92 ± 0.01 [31]. Shown in Table 3, such active contour-based methods have a slightly higher average performance than those of studies that rely on shape priors.

In a study applying DL to aorta segmentation, Noothout et al. used a dilated convolutional neural network for segmentation. To obtain the final segmentation results, the probabilities obtained from the three planes were averaged per class. The Dice coefficients were 0.83 ± 0.07, 0.86 ± 0.06, and 0.88 ± 0.05 for the aorta arch and descending aorta, respectively, and 0.91 ± 0.04 for the aorta [33]. Lartaud et al. segmented multiple cardiac anatomical structures on spectral dual-energy CT images by using a multi-label U-Net, where a Dice coefficient of 0.92 ± 0.02 was obtained for the aorta [34]. However, the DL method relies on effective feature learning of the model or huge training data. Therefore, the aforementioned method has no obvious advantage over the traditional algorithm in terms of performance results. Based on a DL network, this research uses transfer learning through the first stage of the architecture. Thus, the effective learning of the model can be enhanced, and better segmentation performance can be obtained.

Related studies on pulmonary artery segmentation include the following: Moses et al. obtained a high correlation with the manually determined parameters for both mid-cross-sectional area (R = 0.96) and length (R = 0.93) [41]. Xie et al. used the shape before using the cylindrical registration method to segment the pulmonary artery and obtained the mean diameter according to the triangular mesh model and the anatomy label map [38]. Román et al. proposed a 3D convolutional neural network architecture, using realistic deformations to augment data, and obtained a Dice coefficient performance of 0.89 ± 0.07 for pulmonary artery segmentation in CT angiography images [39]. This shows that segmentation of the pulmonary artery remains a challenge even with contrast imaging.

In the aforementioned studies, only one of the segmentation targets required in this study was discussed, which could not meet the needs of this study to be applied to the study of pulmonary hypertension. The following studies have discussed the segmentation of both the aorta and pulmonary artery. Haq et al. established and validated a multi-label DL segmentation model for 2D segmentation for automatic segmentation of 12 cardiopulmonary substructures, including the aorta and pulmonary artery, with segmentation efficiencies of 0.80 ≤ DSC ≤ 0.91 and 0.75, respectively, 0.75 ≤ DSC ≤ 0.94 [35]. Morris et al. used a 3D U-Net to segment multiple structures of the heart and post-processed them using 3D-CRF. The result of the segmentation Dice coefficient of the aorta and pulmonary aorta, collectively called Great Vessels was 0.85 ± 0.03. However, this study requires simultaneous input of CT and magnetic resonance imaging images, which are difficult to obtain simultaneously under normal conditions [36]. Sedghi Gamechi et al. proposed to cut the centerline based on the optimal surface map, and the Dice coefficient of the segmentation result can be obtained as 0.94 ± 0.02 for the pulmonary artery and 0.96 ± 0.01 for the aorta [37]. However, this study is still based on shape priors; therefore, it is easy to encounter the aforementioned problems.

In Table 3, the performance comparison results also show that the segmentation models proposed in this study are superior to other methods in aortic segmentation and only slightly inferior to those of the segmentation algorithms developed based on traditional methods in pulmonary artery segmentation. The method proposed in this study is a DL model; therefore, it is more generalized and robust than traditional methods with good architectural design and training. In the comparison of previous related studies that also used DL models, it can also be seen from Table 3 that the method proposed in this study achieved the best segmentation performance of the aorta and pulmonary artery among the related DL methods.

Several studies have shown a correlation between preoperative pulmonary hypertension and postoperative complications [12,14]. The gold standard approach for the diagnosis of pulmonary hypertension is the direct measurement of pulmonary artery pressure by cardiac catheterization [12,14]. However, this invasive measurement method may not be commonly used for preoperative evaluation of patients with lung cancer. Consequently, owing to the relationship between elevated pulmonary artery pressure and vessel diameter, recent studies have shown the correlation of enlarged pulmonary artery to postoperative complications [42]. However, the method used in the previous studies was 2D measurement of single-cut axial view contrast-enhanced computed tomography image. Automatic 3D segmentation method for both the aorta and pulmonary artery on CT images to accurately calculate the mean 3D diameter has not been reported before. Our proposed model may automatically provide 3D diameter information of the aorta and pulmonary artery before surgery, facilitating the estimation of pulmonary hypertension and supporting preoperative surgical method selection based on the predicted surgical risks.

This study has the following limitations. In the pulmonary artery segmentation model, the contrast enhancement model developed in the first stage of this architecture still needs to be used to provide contrast-enhanced images as inputs for clinical applications. Therefore, this model is more time-consuming and energy consuming than the aorta segmentation model, and the input and model construction methods of this model can be further improved in the future. Second, the types and quantities of data used in this study need to be expanded and increased so that the model in this study can achieve more effective generalization capabilities and improve the applicability of this study model on various non-contrast-enhanced CT images, such as low-dose CT. Third, there are still many artificial parameter settings in the calculation of the diameters of the two blood vessels, which can be further improved into a more automated extraction method.

## 5. Conclusions

To overcome the difficulty of segmenting non-imaging CT images of the aorta and pulmonary artery, this study proposes a two-stage DL segmentation architecture consisting of a contrast enhancement model and segmentation model. This method uses transfer learning to enhance the performance of the segmentation model. The DL method proposed in this study can efficiently complete the segmentation of the aorta and pulmonary artery. Compared with previous research methods for aorta and pulmonary artery segmentation, this study can achieve a high level of segmentation performance. In conclusion, the proposed model may provide the 3D diameter information of two arteries before surgery, facilitating the estimation of pulmonary hypertension and supporting the preoperative surgical method selection based on the predicted surgical risks.

## Figures and Tables

**Table 1:** Hyperparameters of the two types of models used in the proposed architecture.

AA and PA	Learning Rate	Decay	Epochs	Loss Function	Spatial Dropout 3D	Convolution Kernel Size	Activation Function	Output Layer Activation Function
Contrast enhancement model	$10^{-2}$	$10^{-6}$	500	Combination of MAE and DSSIM	0.25	3 × 3 × 3	ReLU	Sigmoid
Segmentation model				Dice loss function				

**Table 2:** Segmentation performance of the two-stage segmentation architecture.

Aorta		Pulmonary Artery	
Model	DSC	Model	DSC
1-AA	0.97 ± 0.007	1-PA	0.91 ± 0.002
		2-PA	0.93 ± 0.002
3D U-Net	0.87 ± 0.025	3D U-Net	0.87 ± 0.0004

1-AA, aorta segmentation model; 1-PA, one-channel pulmonary artery segmentation model by inputting non-contrast-enhanced image; 2-PA, two-channel model by inputting non-contrast-enhanced image and enhanced image; DSC, Dice similarity coefficient stage.

**Table 3:** Comparison of segmentation performance between the method in this research method and those in previous research.

	Method	DSC
Aorta	2016 Jang et al. [25]	0.95 ± 0.02
	2009 Išgum et al. [26]	0.87 ± 0.03
	2012 Kurugol et al. [27]	0.93 ± 0.01
	2013 Avila-Montes et al. [28]	0.88 ± 0.05
	2017 Dasgupta et al. [29]	0.88 ± 0.06
	2014 Xie et al. [30]	0.93 ± 0.01
	2015 Kurugol et al. [31].	0.92 ± 0.01
	2019 Gamechi et al. [32]	0.95 ± 0.01
	2018 Noothout et al. [33]	0.91 ± 0.04
	2021 Lartaud et al. [34]	0.92 ± 0.02
	2020 Haq et al. [35]	0.75 ≤ DSC ≤ 0.94
	2020 Morris et al. [36]	0.85 ± 0.03
	2021 Sedghi Gamechi et al. [37]	0.96 ± 0.01
	Proposed method	0.97 ± 0.007
Pulmonary artery	2015 Xie et al. [38]	0.88
	2018 López-Linares et al. [39]	0.89 ± 0.07
	2020 Haq et al. [35]	0.80 ≤ DSC ≤ 0.91
	2020 Morris et al. [36]	0.85 ± 0.03
	2021 Sedghi Gamechi et al. [37]	0.94 ± 0.02
	Proposed method	0.93 ± 0.002

## Supplementary Materials

The following supporting information can be downloaded at: https://www.mdpi.com/2075-4418/12/4/967/s1, Table S1: Patient clinic pathological features and preoperative results.
