# Correction: Wang et al. Automated 3D Segmentation of the Aorta and Pulmonary Artery on Non-Contrast-Enhanced Chest Computed Tomography Images in Lung Cancer Patients. *Diagnostics* 2022, *12*, 967

**DOI:** 10.3390/diagnostics12081867

**Published:** 2022-08-02

**Authors:** Hao-Jen Wang, Li-Wei Chen, Hsin-Ying Lee, Yu-Jung Chung, Yan-Ting Lin, Yi-Chieh Lee, Yi-Chang Chen, Chung-Ming Chen, Mong-Wei Lin

**Affiliations:** 1 Department of Biomedical Engineering, College of Medicine and College of Engineering, National Taiwan University, Taipei 106, Taiwan; d04548013@ntu.edu.tw (H.-J.W.); f04548034@ntu.edu.tw (L.-W.C.); r08548005@ntu.edu.tw (Y.-J.C.); r08548047@ntu.edu.tw (Y.-T.L.); scsnake@gmail.com (Y.-C.C.); 2 Department of Medicine, National Taiwan University, Taipei 100, Taiwan; b04401128@ntu.edu.tw (H.-Y.L.); b06401027@ntu.edu.tw (Y.-C.L.); 3 Department of Surgery, National Taiwan University Hospital and National Taiwan University College of Medicine, Taipei 100, Taiwan

Errors occurred in the standard deviation of the Dice similarity coefficient (DSC) described in the original publication [1]. A correction of standard deviation value has been made to abstract, results (Section 3.3, second paragraph), discussion (Section 4, first paragraph and fifth paragraph), as well as Tables 2 and 3.

## Text Correction

In abstract, 0.97 ± 0.66 should be corrected to 0.97 ± 0.007, and 0.93 ± 0.16 should be corrected to 0.93 ± 0.002.

In Section 3.3. Segmentation Model, in the second paragraph, 0.97 ± 0.66 should be corrected to 0.97 ± 0.007, and 0.93 ± 0.16 should be corrected to 0.93 ± 0.002.

In Section 4. Discussion, in the first paragraph, 0.97 ± 0.66 should be corrected to 0.97 ± 0.007, 0.91 ± 0.16 should be corrected to 0.91 ± 0.002, 0.93 ± 0.16 should be corrected to 0.93 ± 0.002. In the fifth paragraph, 0.92 ± 0.01 28 should be corrected to 0.92 ± 0.01.

## Table Correction

In the third to fifth lines of Table 2, the DSC value of 0.97 ± 0.66, 0.87 ± 2.46, 0.91 ± 0.16, 0.93 ± 0.16, and 0.87 ± 0.04 should be corrected to 0.97 ± 0.007, 0.87 ± 0.025, 0.91 ± 0.002, 0.93 ± 0.002, and 0.87 ± 0.0004, respectively.

In the 15th and 21st lines of Table 3, the DSC value of 0.97 ± 0.66 should be corrected to 0.97 ± 0.007, and 0.93 ± 0.16 should be corrected to 0.93 ± 0.002.

**Table 2:** Segmentation performance of the two-stage segmentation architecture.

Aorta		Pulmonary Artery	
Model	DSC	Model	DSC
1-AA	0.97 ± 0.007	1-PA	0.91 ± 0.002
		2-PA	0.93 ± 0.002
3D U-Net	0.87 ± 0.025	3D U-Net	0.87 ± 0.0004

1-AA, aorta segmentation model; 1-PA, one-channel pulmonary artery segmentation model by inputting non-contrast-enhanced image; 2-PA, two-channel model by inputting non-contrast-enhanced image and enhanced image; DSC, Dice similarity coefficient stage.

**Table 3:** Comparison of segmentation performance between the method in this research method and those in previous research.

	Method	DSC
Aorta	2016 Jang et al. [25]	0.95 ± 0.02
	2009 Išgum et al. [26]	0.87 ± 0.03
	2012 Kurugol et al. [27]	0.93 ± 0.01
	2013 Avila-Montes et al. [28]	0.88 ± 0.05
	2017 Dasgupta et al. [29]	0.88 ± 0.06
	2014 Xie et al. [30]	0.93 ± 0.01
	2015 Kurugol et al. [31].	0.92 ± 0.01
	2019 Gamechi et al. [32]	0.95 ± 0.01
	2018 Noothout et al. [33]	0.91 ± 0.04
	2021 Lartaud et al. [34]	0.92 ± 0.02
	2020 Haq et al. [35]	0.75 ≤ DSC ≤ 0.94
	2020 Morris et al. [36]	0.85 ± 0.03
	2021 Sedghi Gamechi et al. [37]	0.96 ± 0.01
	Proposed method	0.97 ± 0.007
Pulmonary artery	2015 Xie et al. [38]	0.88
	2018 López-Linares et al. [39]	0.89 ± 0.07
	2020 Haq et al. [35]	0.80 ≤ DSC ≤ 0.91
	2020 Morris et al. [36]	0.85 ± 0.03
	2021 Sedghi Gamechi et al. [37]	0.94 ± 0.02
	Proposed method	0.93 ± 0.002

The authors apologize for any inconvenience caused and state that the scientific conclusions are unaffected. This correction was approved by the Academic Editor. The original publication has also been updated.
