# Supplementary material for: Automated 3D Segmentation of the Aorta and Pulmonary Artery on Non-Contrast-Enhanced Chest Computed Tomography Images in Lung Cancer Patients
Source: Diagnostics (Basel). 2022 Apr 12;12(4):967. doi: 10.3390/diagnostics12040967 (PMC9032785; doi:10.3390/diagnostics12040967)
Supplement: Supplementary file 1 [file diagnostics-12-00967-s001.zip › diagnostics-1637409-supplementary.pdf]

Supplementary Table S1. Patient clinicopathological features and preoperative results

	N (%) or mean ± SD
Total patient number	179
Age, year	60.9±9.4
Female	115 (64.2%)
Smoking	38 (21.2%)
ECOG	
0	23 (12.8%)
≥1	156 (87.2%)
PFT	
FVC, %[a]	108.7±14.7
FEV1, %[a]	108.2±17.2
Lung cancer family history	36 (20.1%)
Histology	
Non-adenocarcinoma[a]	16 (8.9%)
Adenocarcinoma	163 (91.1%)
Pathological stage	
IA	103 (57.5%)
IB	20 (11.2%)
II	36 (20.1%)
III	20 (11.2%)
Post-op ICU stay, day	0.3±0.7
Post-op hospital stay, day	5.3±3.7
Postoperative complications	
All complications	111 (62.0%)
Grade 3a or greater	11 (6.2%)
Grade 3b or greater	0 (0%)
30-day mortality	0 (0%)

ECOG, Eastern Cooperative Oncology Group performance status; FEV1, forced expiratory volume in 1 second; FVC, forced vital capacity; ICU, intensive care unit; PFT, pulmonary function test; SD, standard deviation.
